# Treatment of protruding osseo integrated dental implant

**DOI:** 10.4103/0972-124X.65440

**Published:** 2010

**Authors:** Aravind Buddula, Phillip Sheridan, Ayman Balshe

**Affiliations:** Department of Dental Specialties, Mayo Clinic, Rochester, Minnesota

**Keywords:** Dental implant, osseointegration, perforation

## Abstract

Titanium dental implants have been used in the treatment of partial or complete edentulism. The height and width of the residual alveolus and surrounding anatomical structures can determine the proper position and path of insertion of dental implants. The following case report describes the treatment of a malpositioned osseo integrated dental implant with an apex perforating the buccal cortex of alveolar bone. A 61-year-old male was referred by his local dentist for the chief complaint of a swelling at site of tooth 14 where an implant was present. Intraoral clinical examination revealed an implant supported porcelain fused to metal crown replacing the maxillary right first premolar. A peri-apical radiograph of the implant revealed no signs of peri-implant bone loss or radiolucency. Surgical exploration and modification of the protruding implant. The area healed uneventfully without the need of explantation of the implant in site of tooth 14. We felt that the conservative treatment provided was prudent and treatment of choice and anticipate that the implant will most likely continue to function for a lifetime.

## INTRODUCTION

For over 30 years, titanium dental implants have been used in the treatment of partial or complete edentulism.[1–9] Implant stability is attributed to its anchorage in the surrounding alveolar bone, referred to as osseo integration.[10,11] Proper implant placement in bone is necessary for the success of osseo integration and function.[12] The height and width of the residual alveolus and surrounding anatomical structures can determine the proper position and path of insertion of dental implants. The following case report describes the treatment of a malpositioned osseo integrated dental implant with an apex perforating the buccal cortex of alveolar bone.

## CASE REPORT

In February 2007, a 61-year old male was referred by his local dentist for the chief complaint of a swelling at site of tooth 14, where an implant was present. In December 2002, the patient’s maxillary right first premolar was extracted. A Nobel Biocare TiUnite dental implant was used to replace the missing tooth in February 2003. Since the time of implant placement, the patient noted swelling in the buccal gingival area where the implant was placed. He could feel it with his tongue and fingers. He had always been slightly uncomfortable with this. He preferred that his gingival tissue be returned to its previous form.

**Figure 1:**
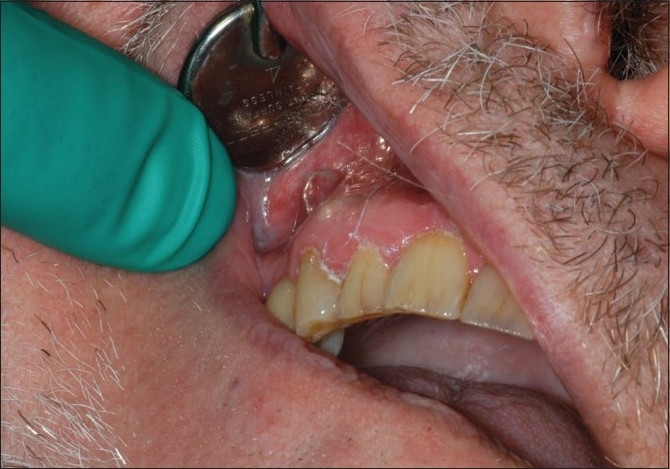
Raised area in site of dental implant

Intraoral clinical examination revealed an implant supported porcelain fused to metal crown replacing the maxillary right first premolar. A peri-apical radiograph of the implant revealed no signs of peri-implant bone loss or radiolucency. Periodontal examination revealed stability of the implant with probing depths ranging from 2-3 mm. There were no clinical signs of peri-implantitis. There was, however, a 5 × 5 mm raised area in the non-keratinized soft tissue facial to the implant [Figure 1]. There were no signs of erythema or infection. Upon digital palpation, there was no soft tissue drainage and the raised area was found to be firm and screw threads could be detected. The patient did not experience any discomfort upon percussion. Hard and soft tissue examination of the rest of the mouth was normal. We recommended surgical exploration and modification of the protruding implant. The patient consented to the treatment plan

**Figure 2:**
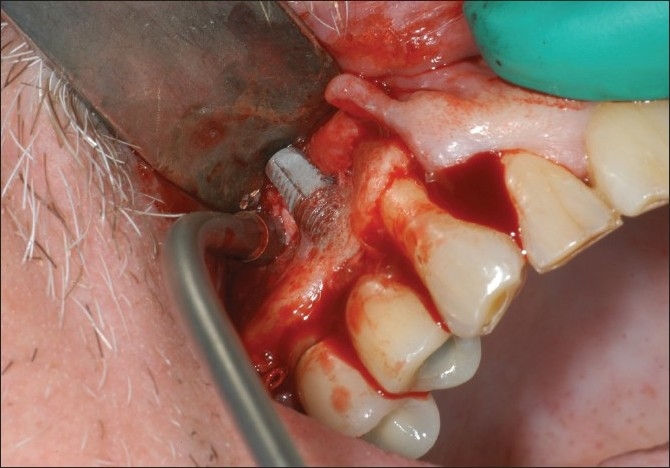
Protruded dental implant

**Figure 3:**
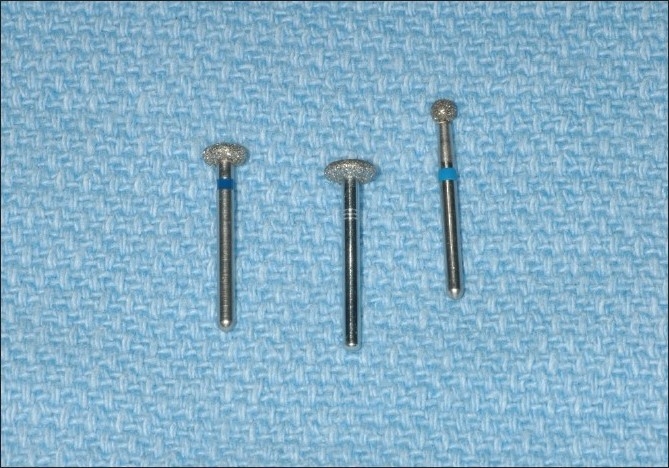
Burs used for the surgery

**Figure 4:**
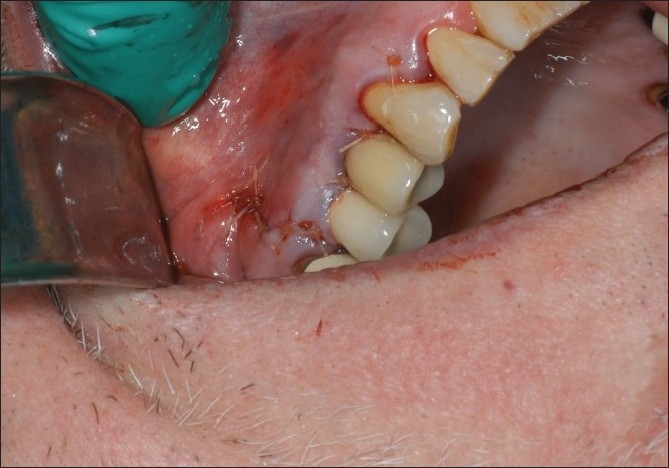
Sutures placed at surgical site

**Figure 5:**
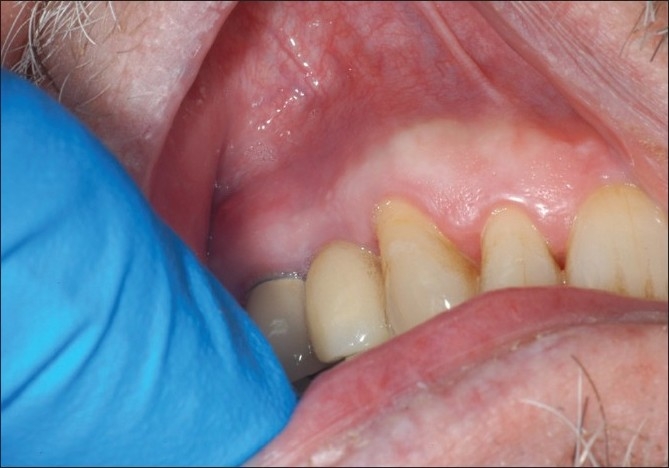
Postoperative healing after 4 weeks

**Figure 6:**
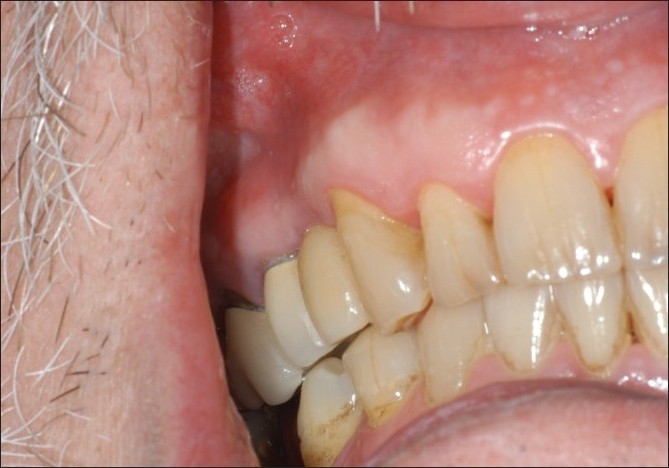
Postoperative healing after 3 months

**Figure 7:**
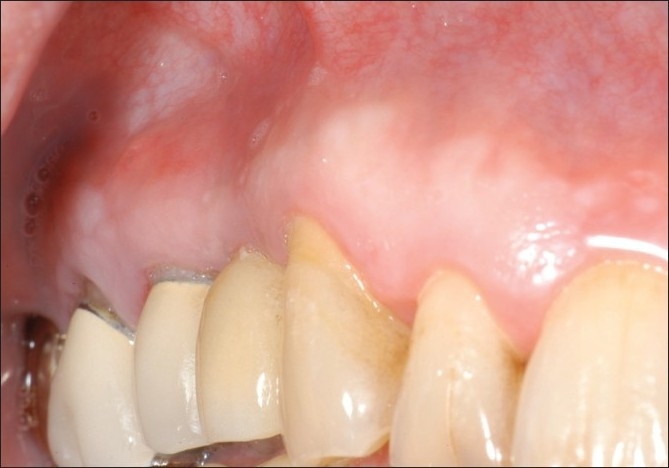
Postoperative healing after 6 months

The surgery was performed under local anesthesia. Sulcular incisions were made extending from the mesio-buccal of the right maxillary first molar distally to the mesio-labial of the right maxillary canine. A vertical releasing incision was also made at the mesio-labial aspect of the right maxillary canine. A full thickness buccal flap was raised. The area of the implant and surrounding alveolar bone was exposed. It was apparent that the apical portion of the dental implant had perforated cortex of the alveolar bone [Figure 2]. The implant was osseo integrated. There was no necrotic bone surrounding the implant. A high speed bur was used to remove the portion of the implant protruding through the buccal plate of bone. A round diamond bur was used to flatten the implant and try to make it flush with the buccal plate of bone [Figure 3]. Copious irrigation was utilized. The facial flap was then repositioned and secured with 4-0 gut suture [Figure 4]. The patient was seen at four weeks [Figure 5], three months [Figure 6] and six months [Figure 7]. The area healed uneventfully.

## DISCUSSION

The authors felt that this case report provided the opportunity to briefly discuss two issues: 1) the appropriate placement of dental implants and 2) treatment options for the malpositioned and symptomatic implants. Recently, there has been much discussion on image-guided implant placement and “flapless” surgery. In this instance, the authors suspected that the clinician placing the implant used “flapless” surgery for the simple flap utilized to treat the problem if used at implant placement would have avoided the problem. Image-guided implant placement is a valuable tool, but most likely not necessary in the majority of cases of implant placement. In the case of implant placement at this No. 14 site, a simple flap would have revealed the concavity in the facial aspect of the alveolus and allowed implant placement while saving the patient lot of expenditure and unnecessary radiation exposure associated with CT scans and image-guided implant placement.

## CONCLUSION

The options considered in treatment of the malpositioned implant were removal of the implant or reduction and reshaping of the protruding portion of the implant. The trauma, cost, and time involved in implant removal and replacement could not be justified. We felt that the conservative treatment provided was prudent and treatment of choice and anticipate that the implant is most likely continue to function for a lifetime.
